# Oxidative Stress and Renal Fibrosis: Recent Insights for the Development of Novel Therapeutic Strategies

**DOI:** 10.3389/fphys.2018.00105

**Published:** 2018-02-16

**Authors:** Wenshan Lv, George W. Booz, Fan Fan, Yangang Wang, Richard J. Roman

**Affiliations:** ^1^Department of Pharmacology and Toxicology, University of Mississippi Medical Center, Jackson, MS, United States; ^2^Department of Endocrinology and Metabolism, The Affiliated Hospital of Qingdao University, Qingdao, China

**Keywords:** chronic kidney disease, antifibrotic therapy, oxidative stress, traditional Chinese medicines, kidney function

## Abstract

Chronic kidney disease (CKD) is a significant worldwide healthcare problem. Regardless of the initial injury, renal fibrosis is the common final pathway leading to end stage renal disease. Although the underlying mechanisms are not fully defined, evidence indicates that besides inflammation, oxidative stress plays a crucial role in the etiology of renal fibrosis. Oxidative stress results from an imbalance between the production of free radicals that are often increased by inflammation and mitochondrial dysfunction, and reduced anti-oxidant defenses. Several studies have demonstrated that oxidative stress may occur secondary to activation of transforming growth factor β1 (TGF-β1) activity, consistent with its role to increase nicotinamide adenine dinucleotide phosphate (NADPH) oxidase (Nox) activity. A number of other oxidative stress-related signal pathways have also been identified, such as nuclear factor erythroid-2 related factor 2 (Nrf2), the nitric oxide (NO)-cyclic guanosine monophosphate (cGMP)-cGMP-dependent protein kinase 1-phosphodiesterase (cGMP-cGK1-PDE) signaling pathway, and the peroxisome proliferator-activated receptor gamma (PPARγ) pathway. Several antioxidant and renoprotective agents, including cysteamine bitartrate, epoxyeicosatrienoic acids (EETs), and cytoglobin (Cygb) have demonstrated ameliorative effects on renal fibrosis in preclinical or clinical studies. The mechanism of action of many traditional Chinese medicines used to treat renal disorders is based on their antioxidant properties, which could form the basis for new therapeutic approaches. This review focuses on the signaling pathways triggered by oxidative stress that lead to renal fibrosis and provides an update on the development of novel anti-oxidant therapies for CKD.

## Introduction

The incidence and prevalence of chronic kidney disease (CKD) continues to increase world-wide, even though some studies suggest that the overall prevalence may be tapering off in some developed countries during the recent decade (Hallan et al., 2016; Murphy et al., 2016; Shin and Kang, 2016). However, according to the United States Renal Data System 2017 Annual Data Report (https://www.usrds.org/adr.aspx), the burden of kidney disease in the USA remains high with rates of kidney failure that require dialysis or transplantation among the highest in the world. The associated poor outcomes result in increased costs to the healthcare system (Mozaffarian et al., 2016). The total annual cost of treating end-stage renal disease in the USA was nearly $30 billion in 2012, representing close to 6% of the total Medicare budget. In addition, a study of nearly 20,000 ethnic Chinese adult women and men showed that changes in renal function, along with metabolic syndrome, predict longevity (Chien et al., 2008). The multivariate relative risk for death from cardiovascular disease was 30.6 in individuals with the highest creatinine and presence of metabolic syndrome compared with those with the lowest creatinine and without metabolic syndrome.

**Figure 1:**
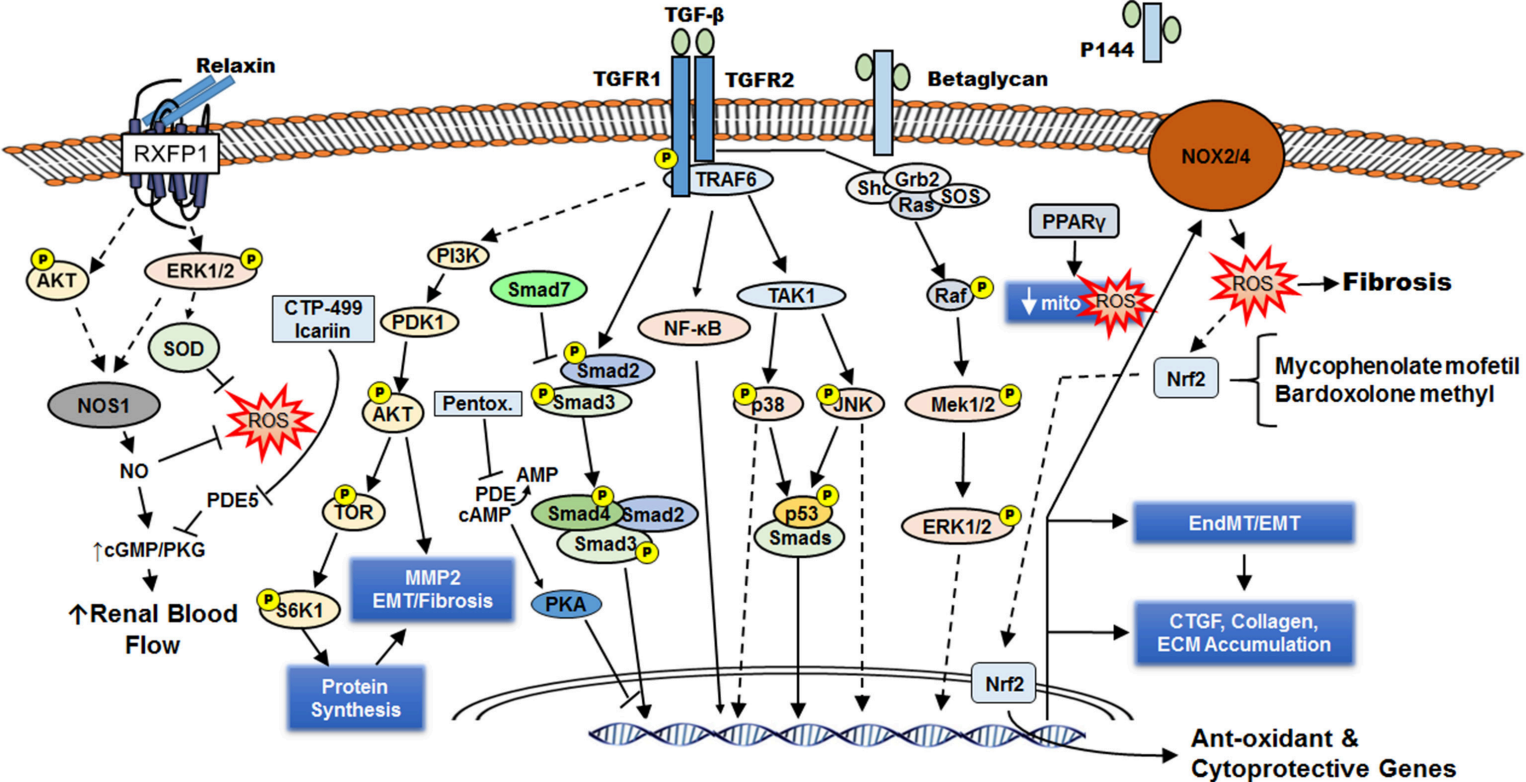
Renal fibrosis occurs downstream of TGF-β intracellular signaling and increased ROS generation. Through its cell surface receptors TGFR1 and TGFR2, TGF-β activates several signaling cascades (including AKT, Smad2/3, NF-κB, p38 MAPK, JNK, ERK1/2) that lead to formation of activated fibroblasts (directly from interstitial fibroblasts or by epithelial mesenchymal transition/EMT or endothelial to mesenchymal transition/EndMT). These myofibroblasts synthesize ECM components leading to fibrosis and express NOX2/4 that generates ROS, which can enhance fibrosis or induce anti-oxidant/cytoprotective genes by activating the transcription factor Nrf2. Activation of the nuclear receptor PPARγ also reduces ROS formation by improving mitochondrial function and defenses. The cell-surface chondroitin sulfate/heparan sulfate proteoglycan betaglycan facilitates the interaction of TGF-β with TGFR2, and a soluble truncated form of betaglycan (P144) can serve as a decoy receptor to attenuate TGF-β signaling. The hormone relaxin engages the G-protein receptor RXFP1 to induce NOS1 expression and generate NO, which can directly reduce ROS or indirectly reduce ROS *via* increased renal blood flow. The PDE5 inhibitors CTP-499 and Icariin enhance the beneficial effects of NO on the kidney. By increasing cAMP and PKA activity, the PDE inhibitor pentoxifylline interferes with Smad3/4-dependent CTGF transcription. CTGF enhances TGF-β signaling.

Increased renal interstitial fibrosis, vasculopathy, tubular atrophy, glomerulosclerosis, and a reduced capacity for renal regeneration are characteristics of CKD. Histologically, progressive kidney disease is marked by renal fibrosis, although underlying mechanisms are incompletely understood (Khwaja et al., 2007; Liu, 2011; Yu et al., 2016). The kidney relies on aerobic metabolism and oxidative phosphorylation for energy production necessary for tubular reabsorption. Many features of renal fibrosis result from the loss of ATP production due to mitochondrial dysfunction, which is associated with increased free radical generation and oxidative stress. Thus, oxidative stress, involving various reactive oxygen and nitrogen species, has an important role in the pathophysiology of CKD (Mimura et al., 2010; Soetikno et al., 2013; Trujillo et al., 2013; Decleves and Sharma, 2014; Kim et al., 2014, 2015). Under normal conditions, the production of reactive species activates many beneficial signaling pathways that maintain homeostasis, but excess production of reactive species is highly damaging. Progressive mitochondrial damage results in loss of efficiency of the electron transport chain, further elevations in ROS generation and lowered ATP production. Hemodialysis and CKD patients exhibit impaired mitochondrial respiration indicative of dysregulated aerobic metabolism and increased oxidative stress (Granata et al., 2009). Disturbances in cellular anti-oxidant systems also impact on downstream signaling events during the pathogenesis of CKD, contributing to renal senescence and cell apoptosis, renal fibrosis, and decreased regeneration of renal cells. Moreover, many traditional Chinese medicine long used for the treatment of renal disorders are antioxidants. Here we review the signaling pathways, transcription factors, and sources of ROS that are altered by oxidative stress during the development of renal fibrosis, and provide an update on the development of novel anti-oxidant therapies for CKD (Figure 1).

## NADPH oxidases and renal fibrosis

There are seven members of the NOX family of membrane-bound NADPH oxidases that generate superoxide and/or hydrogen peroxide. NOX activity is increased in different animal models of renal injury, and reduced NOX activity is associated with renal protection in pre-clinical models of CKD (Baltanas et al., 2013; Holterman et al., 2015a,b). Nox1, Nox2, and Nox4, which are expressed in both human and rodent kidneys, have a central role in mediating oxidative stress in CKD and promote vascular inflammation, dysfunction, and fibrosis (Decleves and Sharma, 2014).

The role of Nox1 in pathophysiology of renal injury is incompletely understood; however, Nox1 is thought to contribute to angiotensin II (Ang II)-mediated hypertension (Lee et al., 2015). Nox2 generates superoxide anions, while Nox4 is constitutively active and may directly generate hydrogen peroxide (Holterman et al., 2015a). Nox4 has a renoprotective action in the unilateral ureteral obstruction (UUO) mouse model of CKD (Decleves and Sharma, 2014), and Nox4-deficient mice exhibited elevated tubulointerstitial fibrosis and oxidative stress after obstruction, and reduced levels of antioxidant markers, including hypoxia-inducible factor 1α and Nrf2. However, the function of Nox4 in CKD is not well defined (Nlandu Khodo et al., 2012; Decleves and Sharma, 2014). Treatment with GKT137831, a dual Nox1 and Nox4 inhibitor, reduces inflammation and ROS production in various murine kidney disease models, along with reduced expression of profibrotic markers and vascular adhesion molecules (Gray et al., 2013; Decleves and Sharma, 2014). A randomized, double-blind, placebo-controlled phase II study (Genkyotex Innovation SAS, 2014) to assess the effect of GKT137831 on albuminuria in patients with type 2 *diabetes mellitus* recently completed, but as of November 2017 the results were not available.

Currently, not much is known about the role of Nox5 in kidney disease, as it is only expressed in human kidneys (Holterman et al., 2015b). Transgenic mice that express Nox5 in podocytes exhibit podocyte foot process effacement, albuminuria, and hypertension (Holterman et al., 2014). These changes were aggravated by streptozotocin-induced diabetes (Holterman et al., 2014). Notably, Nox5 expression is also increased in the kidney of diabetic nephropathy patients (Holterman et al., 2014) and may contribute to the development of CKD.

TGF-β1 causes oxidative stress and fibrosis in the kidney in part by activating NADPH oxidases (Baltanas et al., 2013; Decleves and Sharma, 2014; Lee et al., 2015). Previous studies indicated that Smad3-mediated upregulation of Nox4 may contribute to the conversion of fibroblasts to the myofibroblast phenotype in the kidney, as well their expression of an alternatively spliced variant of fibronectin, Fn-ED-A, downstream of ERK1/2 activation (Bondi et al., 2010). Betaglycan (*aka* TGF-β1 type III receptor) is the most abundant TGF-β1 membrane binding protein. It potentiates TGF-β1 binding to the type I and type II signaling receptors. A synthetic peptide (P144), representing the membrane-proximal ligand-binding domain of betaglycan, effectively blocks TGF-β1-induced effects in various animal models. For example, Baltanas and colleagues reported that P144 prevents oxidative stress and inhibits NADPH oxidases in kidneys of spontaneously hypertensive rats (Baltanas et al., 2013). In addition, P144 reduced renal fibrosis and expression of collagen and connective tissue growth factor (CTGF). Thus, P144 may be a potential antifibrotic therapeutic agent in hypertension induced nephropathy (Baltanas et al., 2013).

## Nuclear factor erythroid-2 related factor 2 and renal fibrosis

The transcription factor nuclear factor erythroid-2 related factor 2 (Nrf2) regulates expression of many genes that oppose inflammatory and oxidative damage, including genes that encode thiol molecules, detoxifying enzymes, antioxidant proteins, and stress response proteins. Accumulating evidence indicates that Nrf2 signaling is protective in various models of renal disease (Choi et al., 2014). Moreover, impaired Nrf2 activity and decreased expression of its antioxidant and renoprotective targets have been observed in CKD animal models (Soetikno et al., 2013; Trujillo et al., 2013). Thus, pharmacological interventions that activate Nrf2 signaling may be helpful in protecting against renal dysfunction in CKD (Soetikno et al., 2013; Tapia et al., 2016).

Mycophenolate mofetil (MMF) is the ester prodrug of mycophenolic acid (MPA), a non-competitive, highly selective, and reversible inhibitor of inosine monophosphate dehydrogenase. Recent evidence indicates that MMF protects from renal damage and reduces oxidative stress and renal inflammation secondary to immunosuppression (Morath et al., 2006). Tapia and colleagues demonstrated that MMF preserved Nrf2-Keap1 and renal dopamine antioxidant pathways and prevented functional renal alterations in a reduced renal mass model of CKD (Tapia et al., 2016). Many naturally occurring Nrf2 activators, such as resveratrol, curcumin, sulforaphane, and cinnamic aldehyde, have been shown to reduce renal oxidative stress and slow the progression of CKD (Choi et al., 2014). The renoprotective effects of curcumin are due in part to induction of Nrf2 and prevention of oxidative stress through the inhibition of mitochondrial dysfunction, the preservation of antioxidant enzymes, and the attenuation of inflammation (Soetikno et al., 2013; Trujillo et al., 2013). However, in a recent pilot study on nondiabetic and diabetic CKD patients curcumin had no effect on proteinuria, glomerular filtration rate, lipid profile, or antioxidant enzyme activities and Nrf2 activation. However, the patients were only treated for 8 weeks, which is arguably too short to reach a definitive conclusion. Curcumin did reduce lipid peroxidation in nondiabetic CKD patients and enhanced antioxidant capacity of plasma in diabetic patients (Jimenez-Osorio et al., 2016). One caveat is that the measurements were from blood, which does not necessarily represent the changes in the kidneys.

Synthetic triterpenoid bardoxolone methyl and its analogs are potent activators of the Nrf2 pathway, and clinical trials that included individuals with type 2 diabetes mellitus and stage 3b or 4 CKD demonstrated that it reduced serum creatinine concentrations for up to 52 weeks (de Zeeuw et al., 2013b). However, a phase 3 BEACON trial was terminated because of serious side effects, including increased mortality (De Zeeuw et al., 2013a; de Zeeuw et al., 2013b; Strutz, 2014). Nonetheless, Nrf2 remains an attractive potential drug target for CKD, as shown by the approval of another Nrf2 activator, dimethyl fumarate, for multiple sclerosis. Nrf2 also regulates the expression of multiple drug-metabolizing enzymes and transporters, including ABCC2 (multidrug resistance-associated protein 2) raising the possibility of serious drug interactions (Molnar et al., 2014). Future trials of Nrf2 activators in patients with chronic diseases need to consider potential drug-drug interactions.

## cGMP-cGK1-PDE signaling and renal fibrosis

Accumulating evidence indicates the cyclic guanosine monophosphate (cGMP)-cGMP-dependent protein kinase 1 (cGMP-cGK1) signaling pathway is involved in renal fibrosis (Shen et al., 2016). Nitric oxide (NO) and phosphodiesterase type 5 (PDE5) are key regulators of this pathway, as they stimulate the formation or breakdown of intracellular cGMP, respectively (Zalba et al., 2001; Firoozi et al., 2005). Notably, NO deficiency related to endothelial dysfunction is linked to the development of hypertension, CKD, and renal fibrosis (Morrissey et al., 1996; Moncada and Higgs, 2006; Baylis, 2008).

Recently, relaxin has been reported to have anti-fibrotic renal effects in various animal models (Danielson et al., 2006; Sasser, 2013, 2014). Relaxin, a member of the insulin-like growth factor family of hormones, is used to treat patients with scleroderma (Negri, 2004). Knockout of the relaxin gene in mice was associated with renal fibrosis that was reversed by exogenous relaxin (Samuel et al., 2005). Previous studies indicated that relaxin exerts its renal anti-fibrotic effects by stimulating the formation and release of NO (Leo et al., 2017), and recent studies demonstrated that the ability of relaxin to increase expression of matrix metalloproteinases (MMPs) and inhibit TGF-β signaling are dependent on NO derived from nitric oxide synthase 1 (NOS1) and NO-cGMP signaling in renal myofibroblasts (Mookerjee et al., 2009; Chow et al., 2012; Sasser, 2013).

Relaxin upregulates NOS1 expression in renal myofibroblasts by activating ERK1/2 through the relaxin family peptide receptor 1 (RXFP1) (Chow et al., 2012). Multiple studies have shown that relaxin also increases the production of NO by activating endothelial NOS (eNOS, *aka* NOS3), thereby improving endothelial function and attenuating inflammation (Mcguane et al., 2011; Dschietzig et al., 2012; Collino et al., 2013; Ng et al., 2016; Valle Raleigh et al., 2017). Relaxin may indirectly increase NO bioavailability by decreasing oxidative stress. In different animal models, relaxin increases expression of manganese superoxide dismutase (MnSOD/SOD2), and decreases 8-hydroxy-2′-deoxyguanosine levels and lipid peroxidation (Masini et al., 1997, 2006). In Ang II-dependent hypertension, relaxin also resulted in reduced circulating and urinary markers of oxidative stress, and nitrotyrosine levels in the kidney (Sasser et al., 2011). Chow et al. (2014) found that the Ang II type 2 receptor (AT_2_) may contribute to the renoprotective effects of relaxin. Relaxin's antifibrotic effects were found to depend on heterodimerization of AT_2_ and RXFP-1 (Chow et al., 2014). Current trials of recombinant human relaxin are assessing its utility in cardiovascular pathologies (Sasser, 2013). Based on the fact that relaxin and its receptor are expressed in the kidney, exploiting the relaxin signaling pathway may have potential for treating fibrotic kidney diseases (Sasser, 2013, 2014).

The nonspecific phosphodiesterase inhibitor pentoxifylline, often used for treating neuropathic injuries and peripheral vascular diseases, inhibited TGF-β1-induced CTGF expression and reduced collagen type I and α-smooth muscle actin expression in normal renal cells (Lin et al., 2005). CTGF binds TGF-β1 and enhances its signaling. Pentoxifylline inhibited CTGF expression by protein kinase A-mediated interference of Smad3/4-dependent CTGF transcription. In the UUO model of renal injury, pentoxifylline attenuated tubulointerstitial fibrosis, accumulation of myofibroblasts, and the expression of CTGF and Col I (α1). There have been conflicting findings concerning its effect on proteinuria (Perkins et al., 2009). CTP-499 is a novel multi-subtype selective, oral inhibitor of phosphodiesterases that is well tolerated (Sabounjian et al., 2016). A recent clinical trial of CTP-499 in CKD patients established its safety and tolerability, justifying further assessment of efficacy with longer-term dosing (Sabounjian et al., 2016). Treatment of alloxan-induced diabetic rabbits with the oral PDE5 inhibitor, vardenafil, normalized serum creatinine levels (Lau et al., 2007). In diabetic rats, sildenafil ameliorated macrophage infiltration into the tubulointerstitium. These preclinical studies demonstrate the therapeutic potential of PDE5 inhibition to reduce oxidative stress and renal fibrosis (Jeong et al., 2009). A recently completed placebo-controlled phase II trial involving patients with diabetic nephropathy tested the hypothesis that the PDE5 inhibitor PF-00489791 (Pfizer) would enhance relaxation of blood vessels in the kidney and subsequently reduce blood pressure and improve renal function (Pfizer, 2013). PF-00489791, which was generally well tolerated, reduced the urinary albumin-to-creatinine ratio indicating improved renal function (Scheele et al., 2016).

Icariin, the major active compound of Epimedium, is a strong PDE5 inhibitor. Dell'Agli et al. (2008) modified the structure of icariin to create icaritin, which increased its potency ability to inhibit PDE5A1 by 80-fold. Icariin is renoprotective in various animal models (Qi et al., 2011; Talapanti et al., 2015; Zhang F. et al., 2015), but it remains to be determined if its beneficial effects are related to enhanced cGMP signaling. More research is needed to assess the effects of icariin specifically for treating kidney fibrosis based on its ability to inhibit PDE5.

## Renal fibrosis and peroxisome proliferator-activated receptor gamma (PPARγ)

PPAR-γ is a member of the ligand-activated nuclear transcriptional superfamily that is expressed in the kidney. In addition to enhancing insulin sensitivity and glucose metabolism, agonists of PPARγ have other effects, such as cell cycle regulation, attenuating inflammation, regulating cytokine production, and decreasing fibrosis. The PPAR-γ agonist pioglitazone protected against renal injury and decreased glomerulosclerosis and interstitial fibrosis in alloxan-induced diabetic rabbits and aged rats. It reduced oxidative stress and improve mitochondrial function in addition to suppressing TGF-β1-induced fibronectin mRNA expression (Gumieniczek, 2003; Yang et al., 2009). L-carnitine has efficacy against hypertension-associated renal fibrosis from *in vivo* and *in vitro* studies. Its effects seem to be dependent on the PPAR pathway as its antifibrotic effects were blocked by the PPAR-γ antagonist GW9662 (Zambrano et al., 2014).

Pioglitazone attenuates fibrosis in part by reducing mitochondrial ROS. It affects mitochondrial function by several means, including (a) by increasing mtDNA; (b) by affecting coupling-uncoupling dynamics and increasing glucose use, thus reducing ROS production; (c) by promoting mitochondrial biogenesis along with PPAR coactivator 1α and nuclear respiratory factor 1; (d) by increasing expression of mitochondrial enzymes involved in eliminating or producing ROS; (e) by reducing cytochrome C oxidase activity and subsequent phosphorylation of p66^Shc^, which induces mitochondrial permeability transition pore opening and ROS. In cells that express p66^Shc^, protein kinase C-β overexpression causes mitochondrial fragmentation and Ca^2+^ signaling defects, which are prevented by PPARγ agonists. This action is likely due to activation of 5′AMP-activated protein kinase (AMPK) and diacylglycerol kinase, two upstream inhibitors directly induced by PPARγ agonists.

The antifibrotic effects of PPARγ agonists are more complicated. PPARγ agonists may also oppose the reduction in NO and TNF-α production *via* PPARγ-independent mechanisms (Zhang et al., 2008; Yang et al., 2009). For example, besides being a partial agonist of PPARγ, telmisartan is also an Ang II type 1 receptor blocker. Hepatocyte growth factor (HGF) is a well-known antifibrotic factor that is the downstream effector of PPARγ activation by telmisartan (Kusunoki et al., 2012). Telmisartan significantly increased renal HGF expression after UUO in Ang II type 1 receptor-deficient mice, and a HGF neutralizing antibody reduced the renal protective action of telmisartan (Kusunoki et al., 2012).

The PPAR-γ agonist rosiglitazone, an antidiabetic drug in the thiazolidinedione class that increases insulin sensitivity in adipocytes, was well tolerated in children with drug-resistant focal segmental glomerulosclerosis (FSGS) in the FONT phase 1 trial. At 16 months of follow-up, 71% of participants showed stable glomerular filtration rate and reduced proteinuria. However, the trial was halted after the release of the warnings by FDA about rosiglitazone's adverse cardiovascular effects in elderly type 2 diabetics. Nevertheless, it should be noted that no specific safety concerns were identified in children involved in the FSGS FONT trial (Joy et al., 2009; Malaga-Dieguez et al., 2015). In summary, a large body of evidence supports the anti-inflammatory and anti-fibrotic effects of PPAR-γ agonists in kidney disease.

## Role of anti-oxidative agents in ameliorating renal fibrosis

Several antioxidant and renoprotective agents have renal protective effects in preclinical or clinical studies (Table 1). Cysteamine bitartrate, an antioxidant drug for nephropathic cystinosis, reduced fibrosis in a mouse model of UUO at 14 and 21 days after surgery (Okamura et al., 2014). Renal oxidized protein levels decreased at each time point, suggesting that oxidative stress was reduced by this agent. In fact, cellular generation of ROS was reduced in cultured macrophages treated with cysteamine. Furthermore, treatment of mice with cysteamine reduced mRNA levels of extracellular matrix (ECM) proteins and interstitial α-SMA-positive myofibroblast proliferation, as well as myofibroblast proliferation and differentiation *in vitro* (Okamura et al., 2014). Two week treatment with cysteamine started 10 days after, renal IRI decreased renal fibrosis by 40%. These findings uncovered a previously unrecognized antifibrotic action of cysteamine *via* TGF-β-independent mechanisms that includes attenuation of oxidative stress and myofibroblast responses to kidney injury (Okamura et al., 2014).

**Table 1:** Novel anti-oxidative agents that ameliorate renal fibrosis.

**Agent**	**Target**	**Finding**
Cysteamine bitartrate	Cellular ROS production; may increase intracellular glutathione pools	Decreased renal fibrosis in UUO and IR models
PXS-4728A	Inhibits Semicarbazide-sensitive amine oxidases (SSAOs)	Reduced renal fibrosis in UUO and cisplatin-induced AKI
AUDA/t-TUCB	Inhibitors of soluble epoxide hydrolase (sEH)	Reduced proteinuria-induced epithelial-mesenchymal transition and myofibroblast levels; prevented renal interstitial fibrogenesis in UUO model
Cytoglobin	Scavenges reactive oxygen species (ROS)	Preserved renal function and reduced fibrosis in remnant kidney model

*AKI, acute kidney injury; IR, ischemia-reperfusion; ROS, reactive oxygen species; UUO, unilateral ureteral obstruction*.

Semicarbazide-sensitive amine oxidases (SSAOs) are copper-containing enzymes that oxidatively deaminate primary amines, thereby producing ammonium, hydrogen peroxide, and aldehydes (Wong et al., 2013). SSAOs are type 1 membrane-bound proteins with a distal adhesion domain and a catalytic amine oxidase site proximal to the membrane (Wong et al., 2013). Stolen et al. (2004) reported that SSAO overexpression increased glomerulosclerosis in mice. Lin et al. (2008) reported that serum SSAO levels were correlated with existing biomarkers of progressive renal disease. Moreover, after adjusting for sex, age, and smoking elevated serum SSAO levels were associated with early CKD (Koc-Zorawska et al., 2012). In the UUO model of renal fibrosis, the selective SSAO inhibitor, PXS-4728A, reduced oxidative stress, suppressed pro-inflammatory and profibrotic cytokine secretion, and limited inflammatory cell accumulation and ECM deposition (Wong et al., 2014; Katagiri et al., 2016). Thus, SSAO inhibitors may be promising therapeutics for preventing kidney disease.

Epoxyeicosatrienoic acids (EETs), which are abundant in the kidney, are cytochrome P-450-dependent derivatives of arachidonic acid with anti-inflammatory, anti-oxidant, and fibrinolytic functions (Zhao et al., 2012; Chen et al., 2014; Kim et al., 2015). Their ability to attenuate oxidative stress results from improved kidney function and anti-inflammatory actions (Khan et al., 2014). Serum levels of EETs were found to be decreased in patients with cardiorenal syndrome (Zhang K. et al., 2015). The cytochrome P450 epoxygenase, CYP2J2, converts arachidonic acid into four regioisomeric EETs. Zhao et al. (2012) showed that rAAV-CYP2J2 gene delivery protected the remnant kidney against renal injury by inhibiting fibrosis and apoptosis *via* regulation of the expression of TGF-β1/Smads, mitogen-activated protein kinase (MAPKs), MMPs, and apoptosis-related proteins. Soluble epoxide hydrolase (sEH) also plays an essential role in CKD by hydrolyzing EETs to the corresponding inactive dihydroxyeicosatrienoic acids. Inhibiting sEH with 12-(3-adamantan-1-yl-ureido)-dodecanoic acid (AUDA) reduced EMT associated E-cadherin suppression, phenotypic transition, α-SMA elevation, PI3K-Akt activation, and GSK-3β phosphorylation (Liang et al., 2015). In mouse models of CKD, knockout of sEH increased EET bioavailability and prevented inflammation and renal tubulointerstitial fibrosis by activating PPAR isoforms and downregulating TGF-β1/Smad3, NF-κB, and inflammatory signaling pathways (Imig et al., 1996; Kim et al., 2014). Kim et al. (2015) analyzed the renoprotective effect of a sEH inhibitor, *trans*-4-(4-[3-(4-trifluoromethylphenyl)-ureido]cyclohexyloxy) benzoic acid (*t*-TUCB) in the UUO model. sEH inhibition enhanced levels of EET regioisomers and inhibited UUO-induced upregulated TGF-β1/Smad3 and NF-κB signaling, oxidative stress, tubular injury, and apoptosis, and also downregulated antifibrotic factors (Kim et al., 2015). Therefore, sEH inhibitors and EETs agonists might be potential strategies for reducing renal inflammation, oxidative stress, and blocking EMT and renal fibrosis (Imig, 2005).

Cytoglobin (Cygb), a novel member of the globin superfamily, is expressed by fibroblasts in various organs. Using the remnant kidney model, Mimura et al. (2010) investigated its role in kidney fibrosis and found that it was attenuated by upregulation of the expression of Cygb. Cygb overexpression was associated with reduced deposition of nitrotyrosine in the kidney and urinary excretion of a marker of oxidative stress (Mimura et al., 2010). Additionally, a mutation in the heme moiety of Cygb that impaired its antioxidant properties attenuated its antifibrotic effects, suggesting that Cygb reduces fibrosis by scavenging free radicals. Thus, Cygb protects the kidney against fibrosis by lessening oxidative stress and may represent a good therapeutic target in CKD (Mimura et al., 2010).

## Traditional chinese medicines for treating CKD

**Figure 2:**
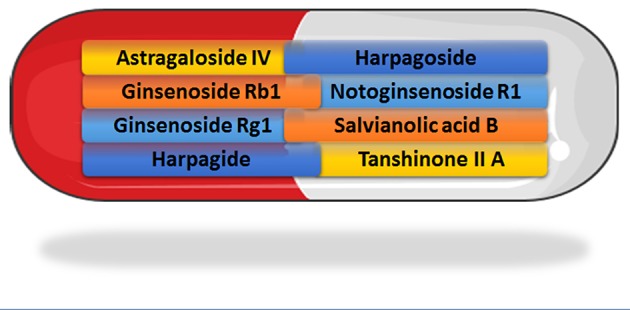
Recently identified constituents of Fufang Xue Shuan Tong (FXST) identified using liquid chromatography–tandem mass spectrometry (Zhou et al., 2015). (Capsule image adapted and reproduced with permission from the copyright holder http://servier.com/Powerpoint-image-bank).

A number of traditional Chinese medicines with anti-oxidant properties have been shown to have beneficial actions in treating CKD in preclinical models, some of which are listed in Table 2. For many years, the traditional Chinese medicine, Fufang Xue Shuan Tong (FXST) capsules, have been used to treat diabetic nephropathy. Eight chemical constituents of FXST were recently identified by periodic polarity-switching liquid chromatography–tandem mass spectrometry (Zhou et al., 2015; Figure 2). In the high-fat diet and low-dose streptozotocin rat model of type 2 diabetes, administration of FXST improved renal function and prevented adverse renal remodeling, while increasing SOD activities and reducing malondialdehyde (MDA) levels (a measure of oxidant status and lipid peroxidation) in the renal cortex (Fang et al., 2012). Thus, it will be important to study each of the components of FXST in isolation and in combinations to identify which of the compounds are the most efficacious agent.

**Table 2:** Traditional Chinese medicines with anti-oxidant and renoprotective actions.

**Medicine/Compound**	**Source**
Berberine	Herbal compound of *Coptis chinensis, Hydrastis Canadensis, Berberis aristata, Berberis aquifolium*, and *Arcangelisia flava*
Curcumin	*Curcuma Longa* L.
Danshen	*Salvia miltiorrhiza (aka* red sage*)*
Fructus crataegi (FC)	Fruit from *Crataegus pinnatifida* Bunge Rosaceae or *Crataegus pinnatifida var. major* N.E.Br.
Fufang Xue Shuan Tong (FXST): SanQi, DanShen, XuanShen and HuangQi	Radix notoginseng, *Salvia miltiorrhiza*, XuanShen, and radix astragali
Hu-Lu-Ba-Wan (HLBW)	*Trigonella foenum-graecum* L. (*TFG*) and *Psoralea corylifolia* L. (*PC*)
Magnesium lithospermate B (MLB/LAB)/tetramer of caffeic acid	*Salviae miltiorrhizae* radix
Radix puerariae (Gegen)	Root of *Pueraria lobata (Willd.)* Ohwi or *Pueraria thomsonii* Benth
RLM/Total flavonoids (TFs)	*Rosa laevigata* Michx.
Rokumi-jio-gan	Hachimi-jio-gan: Rokumi-jio-gan, Cinnamomi Cortex, and Aconiti Tuber; Bakumi-jio-gan: Rokumi-jio-gan contained in Hachimi-jio-gan
Sequoyitol	Found in *Aristolochia arcuata, Amentotaxus yunnan-ensis, Crossostephium chinensi*s, etc.
Sodium ferulate (SF)	Extracted from *Angelica sinensis*, C*imicifuga heracleifolia, Lignsticum chuangxiong*, etc.
Tienchi ginseng or sanchi	*Panax notoginseng*
Total glucosides of peony (TGP)	*Paeonia lactiflora* Pall.
Triptolide/GTW/TwHF	*Tripterygium wilfordii* Hook. F.

Hu-Lu-Ba-Wan (HLBW), a Chinese herbal prescription composed of *Trigonella foenum-graecum* L. (TFG) and *Psoralea corylifolia* L., is used to treat kidney deficiency. HLBW was recently shown to improve hyperglycemia, hyperlipidemia, and proteinuria in type 2 diabetic rats induced by high fat diet and streptozotocin treatment (Zhou et al., 2013). HLBW attenuated ECM accumulation, glomerular expansion and fibrosis, and effacement of foot processes. In addition, HLBW reduced superoxide anion levels, as well as expression and activity of NOX protein. In the same model, a mixture of the Chinese herb Radix Puerariae and Fructus Crataegi, both with strong anti-oxidant actions, attenuated glomerulus mesangial matrix expansion, renal tubular epithelial cell edema, and renal capsule constriction (Chen Z. et al., 2017). Renal fibrosis was attenuated as evidenced by reduced protein levels of α-SMA and collagen IV. Rokumi-jio-gan-containing prescriptions, which are widely used to treat renal dysfunction in Japan, also appear to attenuate oxidative stress in the kidney (Park et al., 2013).

Evidence was recently reported that the ethyl acetate extract of *Salvia miltiorrhiza* (commonly known as Danshen) attenuates oxidative stress in the streptozotocin-treated mouse model of diabetic nephropathy *via* upregulated expression of Nrf2 (An et al., 2017). Triptolide, the main active ingredient of *Tripterygium wilfordii* Hook. F. (TwHF), was found to downregulate expression of oxidative carbonyl proteins in the streptozotocin-treated rat model of diabetic nephropathy (dong et al., 2017). This was associated with improved renal function, decreased kidney nitrotyrosine levels, and increased renal SOD expression. Berberine, a compound of several herbal traditional Chinese medicines, has protective actions on the kidney in several preclinical models, including in streptozotocin–nicotinamide-induced type 2-diabetic mice (Chatuphonprasert et al., 2014). In models of diabetic nephropathy, berberine has been shown to exert anti-fibrotic and anti-inflammatory effects and improve renal function. By blocking NF-κB activation, berberine inhibits rat mesangial cell proliferation and protein expression of fibronectin, TGF-β1, and inflammatory mediators (Jiang et al., 2011; Xie et al., 2013; Lan et al., 2014). Recently, berberine was reported to activate Nrf2 nuclear translocation and inhibit apoptosis of renal tubular epithelial cells induced by high glucose (Zhang et al., 2016). Curcumin from *Curcuma Longa* L. has well-documented anti-oxidant and renoprotective actions in several animal models of kidney injury, including Rhabdomyolysis-induced AKI (Chen X. et al., 2017), maleate-induced renal injury (Molina-Jijon et al., 2016), streptozotocin-induced diabetes (Ho et al., 2016), and the rat remnant kidney (Tapia et al., 2013). Recent evidence indicates that curcumin and its derivatives are potent hydrogen donors and may thus act primarily as chain breaking antioxidants, as opposed to direct free radical scavengers (Morales et al., 2015).

Total glucosides of peony (TGP), extracted from the traditional Chinese herb root of *Paeonia lactiflora* Pall., was reported to prevent diabetes-associated renal damage from oxidative stress in the rat and increase renal activity of antioxidant enzyme such as SOD and catalase (Su et al., 2010). A subsequent study found that TGP improved renal function and attenuated the expression of pro-inflammatory cytokines (Xu et al., 2014). A recent small prospective, randomized, parallel-group study involving 76 type 2 diabetics with diabetic kidney disease found that TGP reduced albuminuria and circulating inflammatory markers (Zhu et al., 2016).

Sodium ferulate (SF), extracted from several plants including *Angelica sinensis*, C*imicifuga heracleifolia*, and *Lignsticum chuangxiong*, has well known anti-platelet, antithrombotic, and antioxidant actions. SF was reported to protect the kidney of streptozotocin-induced diabetic rats by inhibiting the expression of TGF-β1 and collagen IV (Zhao et al., 2004). In a recent large retrospective cohort study involving CKD patients, certain renoprotective Chinese herbal medicines, especially those containing *Angelica sinensis*, were associated with a lower risk of mortality (Hsieh et al., 2017). The compound Sequoyitol present in several plants, including *Aristolochia arcuata, Amentotaxus yunnanensis*, and *Crossostephium chinensi*s, was found to improve insulin levels and renal function of diabetic rats, induced by a high-fat diet and low dose streptozotocin (Li et al., 2014). Renal level of total antioxidative capacity was increased, while ROS and MDA levels were decreased, as was expression of NOX components, NF-κB, and TGF-β1.

Total flavonoids (TFs) from *Rosa laevigata* Michx. (RLM) fruit were found to attenuate ischemia-reperfusion injury in the rat, which was associated with upregulation of Nrf2, as well as silent information regulator factor 2-related enzyme 1 (Sirt1) and heme oxygenase-1 (HO-1) (Zhao et al., 2016). Levels of ROS, MDA, NF-κB p65, and expression of inflammatory cytokines were decreased, while activities of SOD and glutathione peroxidase (GSH-Px), and levels of anti-oxidant glutathione (GSH) were increased. Similar findings were reported in the same model using *Panax notoginseng*, a well-known traditional Chinese herb medicine with antioxidant and anti-inflammatory properties (Liu et al., 2010).

Magnesium lithospermate B (MLB/LAB), extracted from *Salviae miltiorrhizae* radix, is a scavenger of superoxide anions and hydroxyl radicals (Wu et al., 2000). MLB was recently shown to attenuate renal oxidative stress and kidney damage in elderly rats (Park et al., 2017). MLB administration was associated with reduced renal protein expression of major NOX subunits (Nox4 and p22^phox^), as well as NF-κB p65, cyclooxygenase-2, phospho-p38 MAPK, and inducible NOS. MLP was similarly protective against renal injury in streptozotocin-induced diabetic rats (Lee et al., 2003) and in rats after subtotal nephrectomy (Yokozawa et al., 1997).

## Conclusions

In conclusion, oxidative stress plays an important role in the pathophysiology of renal fibrosis. Increased ROS formation is not only a consequence of renal fibrosis, but also positively impacts on pro-fibrotic signaling. A better understanding of the signaling pathways by which oxidative stress induces renal fibrosis may lead to the development of novel therapeutic strategies. The mechanism of action of many traditional Chinese medicines with proven therapeutic value in treating kidney disorders is based on their anti-oxidant properties. A better understanding of the cellular and molecular mechanisms behind their actions will undoubtedly lead to new drugs that effectively prevent or reverse renal fibrosis. It is our contention that further studies on the mechanisms underlying the renoprotective effect of antioxidant agents in traditional Chinese medicines will provide new insights that propel the development of novel therapies to slow the progression of CKD.

## Author contributions

WL conceived the idea and helped research and write the manuscript; GB helped research and write the manuscript; FF helped write the manuscript and offered insight into traditional Chinese medicines; YW helped write the manuscript; RR helped research, write, and edit the manuscript.

### Conflict of interest statement

The authors declare that the research was conducted in the absence of any commercial or financial relationships that could be construed as a potential conflict of interest.
